# Ethical Issues in the Use of Smartphone Apps for HIV Prevention in Malaysia: Focus Group Study With Men Who Have Sex With Men

**DOI:** 10.2196/42939

**Published:** 2022-12-23

**Authors:** Antoine Khati, Jeffrey A Wickersham, Aviana O Rosen, Jeffrey Ralph B Luces, Nicholas Copenhaver, Alma Jeri-Wahrhaftig, Mohd Akbar Ab Halim, Iskandar Azwa, Kamal Gautam, Kai Hong Ooi, Roman Shrestha

**Affiliations:** 1 Department of Allied Health Sciences University of Connecticut Storrs, CT United States; 2 AIDS Program Yale School of Medicine New Haven, CT United States; 3 Department of Physical Therapy University of the Philippines Manila Philippines; 4 Centre of Excellence for Research in AIDS (CERiA) University of Malaya Kuala Lumpur Malaysia

**Keywords:** HIV, mobile health, mHealth, mobile app, HIV prevention, men who have sex with men, privacy, confidentiality, Malaysia, mobile apps, ethics, focus group, implementation, user privacy, mobile phone

## Abstract

**Background:**

The use of smartphone apps can improve the HIV prevention cascade for key populations such as men who have sex with men (MSM). In Malaysia, where stigma and discrimination toward MSM are high, mobile health app-based strategies have the potential to open new frontiers for HIV prevention. However, little guidance is available to inform researchers about the ethical concerns that are unique to the development and implementation of app-based HIV prevention programs.

**Objective:**

This study aimed to fill this gap by characterizing the attitudes and concerns of Malaysian MSM regarding HIV prevention mobile apps, particularly regarding the ethical aspects surrounding their use.

**Methods:**

We conducted web-based focus group discussions with 23 MSM between August and September 2021. Using in-depth semistructured interviews, participants were asked about the risks and ethical issues they perceived to be associated with using mobile apps for HIV prevention. Each session was digitally recorded and transcribed. Transcripts were inductively coded using the Dedoose software (SocioCultural Research Consultants) and analyzed to identify and interpret emerging themes.

**Results:**

Although participants were highly willing to use app-based strategies for HIV prevention, they raised several ethical concerns related to their use. Prominent concerns raised by participants included privacy and confidentiality concerns, including fear of third-party access to personal health information (eg, friends or family and government agencies), issues around personal health data storage and management, equity and equitable access, informed consent, and regulation.

**Conclusions:**

The study’s findings highlight the role of ethical concerns related to the use of app-based HIV prevention programs. Given the ever-growing nature of such technological platforms that are intermixed with a complex ethical-legal landscape, mobile health platforms must be safe and secure to minimize unintended harm, safeguard user privacy and confidentiality, and obtain public trust and uptake.

## Introduction

### Background

Between 2020 and 2021, HIV incidence around the globe witnessed an annual decline of 3.6%, the smallest since 2016 [1]. Although recent HIV incidence and mortality trends have declined globally, some countries in Southeast Asia still bear a disproportionate HIV burden [2-4]. Among the 1.5 million new HIV cases reported globally in 2021, a total of 260,000 were from the Asia and Pacific region, where HIV infection rates are now rising, even in locations where they had been previously declining [1].

Malaysia has one of the highest HIV prevalence in the Asia-Pacific region and remains one of the only a few countries globally where HIV-related mortality has been steadily increasing [5]. In addition, it remains one of the HIV hot spots in that region, with alarming increases in HIV infections [1] and 5500 new cases in 2021 among adults aged ≥15 years [6]. Malaysia’s HIV epidemic is rapidly expanding, with recent evidence suggesting accelerated sexual transmission, especially in men who have sex with men (MSM) [7,8]. Several factors may potentiate HIV transmission among Malaysian MSM, including condomless sex, sexually transmitted infections, and comorbid psychiatric or substance use disorders [9-14].

Same-sex behavior is illegal in Malaysia under the Sharia law and section 377 of the Malaysian Penal Code. Under section 377, “carnal intercourse against the order of nature” is punishable with imprisonment that may last for 20 years and include whipping [15,16]. Similarly, the Islamic Sharia law criminalizes anal sex among Muslims and carries similar punishments with imprisonment, fines, and whipping. Instances of Malaysian authorities raiding venues regularly visited by MSM to enforce the law have also been reported, along with cases of physical and psychological abuse during incarceration among MSM and other men perceived to be MSM who were arrested under section 377 [15].

In this hostile environment that criminalizes and punishes same-sex sexual activity, high levels of stigma and discrimination against Malaysian MSM ensue, including in health care settings [17-19]. The resulting fear of legal repercussions related to the disclosure of same-sex behavior and judgment from health care providers [17,20-22] hinder access to and use of HIV prevention and treatment services, contesting the role of in-person venues for HIV prevention services, such as HIV testing and pre-exposure prophylaxis (PrEP).

The recent advances in wireless technology, along with the change in the traditional health care service delivery model, have led to the development of mobile health (mHealth), which offers an unparalleled opportunity to deliver internet-based health care services. These technologies have increasingly been used in health care management and delivery to address various health conditions and diseases, including diabetes and hypertension management, smoking cessation, weight loss, increased physical activity, and sexually transmitted infections [23-28]. Such technologies have many potential health care benefits, such as monitoring users’ health status remotely and continuously, improving access to health care services, lowering health care costs, increasing patients’ awareness of health status, and improving patient-provider communications.

mHealth interventions can particularly benefit HIV prevention efforts in Malaysia, where smartphone ownership among MSM is nearly universal. Existing data indicate that over 97% of MSM in Malaysia have access to a smartphone, making mHealth a highly feasible platform for this subpopulation [29]. Furthermore, findings from recent studies demonstrate a strong preference among MSM for smartphone apps over other modalities (eg, text, phone calls, and emails) to engage in mHealth HIV prevention tools, thus supporting the development and deployment of smartphone apps for HIV prevention [29-31]. mHealth could serve as an innovative platform to improve access to and use of HIV testing, linkage to PrEP and antiretroviral therapy, and other support services (eg, mental health and substance use).

Despite the numerous benefits of app-based platforms, several ethical challenges accompany their use and must be considered to safeguard user safety, privacy, and rights. These include the protection of privacy and confidentiality, informed consent (including transparency with users about potential risks), data management and careful communication of data, mHealth product regulation and evaluation before public dissemination and uptake, and equity and equitable access [32-34]. Researchers, clinicians, and app developers must be knowledgeable about the ethical issues surrounding the use of HIV prevention apps to improve the perceived usefulness, interpretability, navigability, feasibility, and acceptability of such platforms and tailor them to the specific needs of their target population.

### Objectives

Although ethical considerations for telehealth have been well addressed in the literature, particularly in high-income countries [35,36], patient feedback on ethical issues around using smartphone apps for HIV prevention is sparse, especially in low- and middle-income countries such as Malaysia [31]. Therefore, this study aims to fill this gap by characterizing the attitudes and concerns of Malaysian MSM regarding HIV prevention mobile apps, particularly regarding the ethical aspects surrounding their use.

## Methods

### Study Design

The reporting of this manuscript was guided by the Standards for Reporting Qualitative Research [37]. This study used a qualitative exploratory design using focus group discussions (FGDs) as an interview technique. The authors first developed an interview guide, which was consolidated according to the available literature on key ethical issues related to mHealth [32,33,38].

### Existing Ethical Principles and Interview Guide Development

To generate a comprehensive list of ethical principles related to HIV prevention mHealth app use, we first identified the general ethical framework’s 8 principles for biomedical research [39]. These included collaborative partnership, social value, scientific validity, fair subject selection, favorable risk-benefit ratio, independent review, and informed consent. We then explored ethical issues in the context of mHealth research among people living with HIV [32], which further stressed the importance of associated physical, social, behavioral, and psychological risks and privacy and confidentiality risks. In addition, we identified 2 mHealth ethics frameworks [33,38] that elaborated on the accessibility of mHealth platforms, informed consent, and regulation of mHealth products.

To develop the interview guide, we modified the general ethical framework for biomedical research principles by adapting it to mHealth-related research. For example, the social value and scientific validity principles were combined [32]. The collaborative partnership and independent review ethical principles were not addressed in the mHealth-specific ethics frameworks and were removed from the guide. Furthermore, Carter et al [33] described issues with access to mHealth, such as socioeconomic status and physical or mental impairments. These were subsumed under fair subject selection. Carter et al [33] and Fisher et al [38] discussed privacy and consent in more detail, which helped to populate probing questions. Finally, Shrestha et al [33] discussed regulation, which was not addressed in other frameworks and was thus added to the topic list. Finally, feedback from authors was taken until a consensus was reached. This was followed by 4 iterative rounds of revisions, rearrangements, and merging of topics between coauthors that ultimately generated the final version of the guide used in all FGDs.

### Study Setting and Recruitment

We recruited a convenience sample of 23 MSM between August and September 2021. The eligibility criteria were as follows: (1) a self-reported negative or unknown HIV status, (2) aged ≥18 years, (3) identifying as male, and (4) the ability to read and understand English or Bahasa Malaysia. Participants were recruited using advertisements on geosocial networking (GSN) apps for MSM (ie, Hornet) and Facebook (a popular social networking website for the general population). The GSN apps pushed the advertisement as a message to the chat inboxes of all users in Malaysia. Targeted banner advertisements were used on Facebook. These banners appeared either as a static advertisement on the right-hand pane of the website or an advertisement that resembled a standard post that users could encounter while scrolling through their feed; clicking on the advertisements directed interested persons to another page to provide their contact information. A research staff member then contacted eligible individuals, assessed their willingness to join the study, and shared more details with eligible and willing participants.

### Study Procedures and Measures

Owing to movement restrictions related to the COVID-19 pandemic in Malaysia, participant recruitment and FGDs were conducted via the internet. FGDs were conducted using a videoconferencing platform (ie, WebEx), and each session lasted approximately 90 minutes. Three FGDs were conducted until theoretical saturation was reached [40]. The first 2 FGDs included 8 participants, whereas the last session included 7. A trained facilitator led the FGD session, whereas a cofacilitator took notes, recorded nonverbal cues, and collected chat entries.

Each session began with a brief introduction in which a description of the purpose of the FGD was communicated to participants; time was dedicated to answering participants’ questions and concerns, and participants were reminded that the session would be recorded. Participants were informed that they were not required to use their real names and that they could keep their cameras off during the session to ensure privacy. Participants were encouraged to share their feedback verbally or through the chat function, which was frequently reviewed to ensure that all participant responses were collected. Each session was audio recorded and transcribed. Two authors reviewed the transcripts for accuracy.

Following informed consent, participants completed a brief web-based survey on Qualtrics that included sociodemographic information (eg, age, ethnicity, and sexual orientation), access to communication devices (eg, mobile phones, tablets, and laptops), and awareness of and previous use of HIV prevention services (eg, HIV testing and PrEP).

Before the discussion, participants were briefly provided with information regarding the use of smartphone apps for HIV prevention, including the common features and functions that apps for HIV prevention comprise to support HIV prevention efforts and care. Some key app features included receiving reminders to take PrEP; scheduling appointments with health care providers; ordering PrEP and HIV self-testing kits; and reviewing test results, medication intake, and other health-related information through the app.

Participants were then asked to provide insights into perceived individual- and community-level ethical challenges and concerns about using an HIV prevention app with such features. The FGDs started with a general question to participants regarding their impression and thoughts on the use of a mobile app that contains the features and functions that were just presented (“What is your general impression regarding the use of such an app with these features embedded?”). A more specific question followed, pertaining to the risks of using such apps for HIV prevention (“What do you think are some of the risks of using such an app for HIV prevention efforts?”). Participants were then probed on each of the 5 ethical principles (previously described) that were the focus of the FGD sessions through multiple questions on each ethical aspect.

The first ethical concern was privacy, specifically regarding anonymity and data deidentification. The subconstructs included app visibility (eg, “how would you feel about the app being visible on your phone?”), photos or avatars (eg, “how do you feel about using photo avatars and aliases in setting up your profile in the app?”), and reminders and notifications (eg, “what concerns might you have regarding PrEP reminders?”). The second ethical principle was the third-party use of data. Therefore, questions regarding potential confidentiality breaches were asked (eg, “do you have any concerns about your data being accessible to others?”). Questions regarding the storage and transmission of data were then addressed, including questions about server locations and types (eg, “if the data were stored on a server in Malaysia or in a foreign country, how would you feel about your data security/privacy?”), governmental interception of stored data (eg, “are you concerned about the government accessing your personal information from the app?”), and sharing personal health-related information (eg, “how comfortable do you feel about sharing personal health information via the app?”). Finally, questions about access to mHealth technology (eg, “do you think there are smaller groups in your community who will have difficulty using the app or getting access to a smartphone?”) and regulation of mHealth products in Malaysia (eg, “how do you feel about using an app that does or does not meet certain minimum standards for security, privacy or quality that leading companies or industry groups set?”) were raised. In addition, while each ethical issue was being discussed, participants were encouraged to provide recommendations or suggestions to help mitigate or solve the concerns they brought forth. For example, during the discussion on third-party use of data, participants were asked the following question: “What steps could app developers take to address the security and confidentiality breach concerns that you have?”

### Data Analysis

Descriptive statistics for variables collected via a brief Qualtrics survey were computed, including frequencies and percentages for categorical variables, using SAS (version 9.4; SAS Institute, Inc). The transcripts were analyzed using abductive thematic analysis to inductively identify and interpret the concepts and themes that emerged from the interview transcripts. This method involves multiple readings of transcripts and interview notes and analytic induction via open and axial data coding using Dedoose software (SocioCultural Research Consultants, Los Angeles, CA, US) to organize transcripts thematically. Transcripts were checked for any inconsistencies or mistakes before coding was initiated. A codebook was developed with mutually agreed-upon codes derived from the interview transcripts, and coding was completed independently by 2 researchers (including a senior coder). To ensure reliability, codes were constantly compared for agreement and discussed between the 2 coders, and the senior coder cross-checked all codes [41,42]. The Cohen κ coefficient for agreement was estimated to assess the interrater concordance. Open coding, which involved assigning conceptual codes to small sections of words, phrases, and sentences in transcripts, was followed by axial coding, whereby relationships among similar concepts and categories were identified and combined into themes.

### Ethics Approval

Participants provided verbal consent before starting the FGDs and were informed that participation in the study was voluntary. Participants were compensated with RM 45 (approximately US $10) per person for their participation. The study protocol was approved by the Institutional Review Board at the University of Connecticut (L21-0007). All FGD transcripts were deidentified before the analysis, and the web-based survey data were anonymous.

## Results

### Demographics

Table 1 provides information on participant characteristics. The mean age of the participants was 33.4 (SD 12.0) years. Most (13/23, 57%) participants were Chinese, identified as being gay (21/23, 91%), had daily access to a smartphone with internet (22/23, 96%), and had daily access to the internet (23/23, 100%). In addition, the vast majority (22/23, 96%) had taken an HIV test at least once and had heard of PrEP previously (22/23, 96%). Finally, 61% (14/23) of participants had already taken PrEP in the past.

**Table 1:** Characteristics of participants (N=23).

Variables		Values
Age (years), mean (SD)		33.4 (12.0)
**Ethnicity^a^, n (%)**		
	Chinese	13 (57)
	Malaya	8 (35)
	Indian	2 (9)
**Sexual orientation, n (%)**		
	Gay	21 (91)
	Bisexual	2 (9)
**Access to communication technology^b^, n (%)**		
	Landline	1 (4)
	Mobile phone with internet access (ie, smartphone)	22 (96)
	Mobile phone without internet access	2 (9)
	Tablet	13 (57)
	Laptop	19 (83)
	PC	7 (30)
Had daily access to internet, n (%)		23 (100)
**Primary device to access the internet, n (%)**		
	Smartphone	17 (74)
	Tablet	1 (4)
	Laptop	3 (13)
	PC	2 (9)
**Ever tested for HIV, n (%)**		
	Yes	22 (96)
	No	1 (4)
**Ever heard of PrEP^c^, n (%)**		
	Yes	22 (96)
	No	1 (4)
**Ever taken PrEP, n (%)**		
	Yes	14 (61)
	No	9 (39)

^a^Percentages may not add to 100% owing to rounding.

^b^Percentages may not add to 100% because answers are nonexclusive.

^c^PrEP: pre-exposure prophylaxis.

### Ethical Issues and Concerns Around the Use of HIV Prevention Smartphone Apps

#### Overview

Several themes were identified during FGDs regarding ethical concerns around using mobile apps for HIV prevention. Participants were generally concerned about privacy and confidentiality issues, including uploading personal information to mobile apps and how apps would be visible to others on mobile home screens. Another emerging theme was storage and data ownership, whereby participants raised concerns about data management and storage, including server types and locations and governmental access to data. Moreover, concerns about informed consent, access to web-based communication technology (eg, smartphones), and regulation were raised. Participants noted several subpopulations in Malaysia that might have trouble using or accessing mobile apps and demonstrated preferences for endorsing agencies, quality control checks, and locations of app developers and their affiliations. The major themes (privacy and confidentiality, storage and data ownership, access to mHealth technology, informed consent, and regulation) are discussed below in order of overall importance. Cohen κ coefficient showed strong agreement between the coders (Cohen κ=0.851).

#### Privacy and Confidentiality

The participants highlighted concerns regarding the privacy and confidentiality of the data collected by the app (Table 2). Participants were concerned about third-party access to the data collected via the app (or knowledge of their HIV app use) through incidental discovery, for instance, by someone accessing the phone (eg, family or friends):

Some people have a keen eye, and they might read the app’s name, for example, and guess that, it is related to HIV. Some people would discriminate against others based on their medications, like antiretroviral medications.

**Table 2:** Ethical concerns brought up in focus group discussions regarding privacy and confidentiality (N=23).

Ethical category themes and subthemes		Mentions, n	
**Uploading personal information**		12	
	Information for identity verification	2	
	Uploading HIV test result	10	
	Weary of uploading positive results	3	
	Not concerned with uploading information	6	
**Setting up account**		3	
	Concerns over disclosing personal information	3	
Doctors upholding privacy		2	
**App visibility**		14	
	App icon	8	
	PrEP^a^ stigma within MSM^b^ community	1	
	Visibility is not an issue	5	
**Notifications**		7	
	Discreet or silenced notifications preferred	1	
	Notification customization options	4	
	Pop-up display or message preview	3	
	Sounds or ringtone	5	
**Photos and avatars**		5	
	Avatars are an excellent option for discretion	2	
	Customization options are preferred	3	
	Photos are not an issue for younger MSM	2	
Not worried about privacy		5	
Level of security		5	
**Breach via theft or hacking**		4	
	Access via log-in, password, or OTP^c^	2	
	Not a concern	2	

^a^PrEP: pre-exposure prophylaxis.

^b^MSM: men who have sex with men.

^c^OTP: one-time password.

Although this was a relatively uncommon concern, some participants highlighted the need to ensure that information regarding the identity of the app developers or owners (eg, academic institutions, health care organizations, nongovernmental organizations, pharmaceutical companies, and government agencies) is transparent. For example, one individual mentioned:

I want to know “who owns this company” or whatnot. Suppose I feel it is associated with an academic institution or at least with one established in the health industry. In that case, I think it’ll be safer to upload my personal info on it.

Possible solutions to address privacy and confidentiality issues included a log-in screen on opening the app and multifactor authentication or fingerprint or facial recognition methods:

I wonder, sending out like a multi-factor authentication is quite good.

Privacy concerns regarding app visibility and tailoring notifications and avatars were also discussed. For example, 1 participant pointed out that app visibility should be minimized, suggesting that this “would be great for people who are still not comfortable with their sexuality.” One recommendation to address this issue was to incorporate a discreet app icon to make the app inconspicuous when notifications appear, thereby reducing the risk of accidental disclosure to family members or friends. Another suggestion was to deliver encrypted messages to the app users:

Maybe you could customize [the notifications] so that the app sends a message that says, “It’s 8 am, time to brush your teeth,” and you know, that doesn’t really mean brush your teeth, it means “take your PrEP.”

Another opportunity for discretion within the app, as suggested by the participants, involved the customization of profile photos and avatars. A participant suggested:

I think having the option to have avatars and stuff like that will help them to have the confidence to use the app, so I think it’s a great idea to have a few avatars as an option. For me personally, I wouldn’t be uploading my picture on your app, but I don’t think it really matters to have a personal picture on it.

In general, participants seemed to value discretion within the app, leaving no opportunity for accidental disclosure incidents.

#### Storage and Data Ownership

Another prominent concern that the participants raised was storing and sharing data collected via the app (Table 3). Participants expressly referred to the management of collected data and the data storage methods, including the entities in charge of data storage and individuals and organizations with access to it:

The communication itself is not a problem, but the custodian of the data is. I believe the chat will be safe. I mean, it will be encrypted, right? But it’s just a matter of how the data is being kept.

**Table 3:** Ethical issues and concerns brought up in focus group discussions regarding data storage and ownership (N=23).

Ethical category themes and subthemes		Mentions, n
Data ownership		3
Government access to stored data		3
Data management concerns		34
How will data be used		1
**Data storage**		14
	Location of the server	2
	Reputable operator	3
	Who stores or manages data	3
	Location of the server is not a concern	5
**Data transmission risks**		4
	Avoid public Wi-Fi	1
	Not a concern	2

Many participants expressed concerns that their personal health-related information may become publicly available and accessible by government agencies or that their sexual orientation may be disclosed to others, including health care providers:

I’m concerned about the custodian of the data that’s being collected by this app. Because it is not just a normal app, you will also be collecting data regarding health.

Participants further pointed out the threat to privacy through third-party access of the app data through government interception or hacking of information sent over the internet. For many participants, the underlying concern in this topic involved the likelihood of Malaysian government officials obtaining access to app data.

Interestingly, most participants who raised concerns about data storage servers were not too concerned about the server’s location. The need for reputable server hosting companies was discussed in several instances. Moreover, data transmission risks were not a major concern. Avoiding public Wi-Fi (or other telecommunication networks) was infrequently mentioned, and no other specific transmission risks were raised.

#### Access to mHealth Technology

Participants stressed that there is an ethical imperative to not exclude any individuals at risk for HIV (eg, discreet or hidden MSM subgroups, those with low income, those who reside in rural areas, and those who cannot afford technology) from benefiting from this platform (Table 4):

But then you also need to understand that the living standard itself, you know, people who are from the lower class might not be able to afford all this.

**Table 4:** Ethical issues and concerns brought up in focus group discussions regarding mobile health (mHealth) access and regulation (N=23).

Ethical category themes and subthemes			Mentions, n
**Access to mHealth^a^ technology**			N/A^b^
	**MSM^c^ subpopulations**		10
		College students	3
		Discreet MSM	3
		Low-income MSM	2
		MSM in rural areas	1
		MSM who use drugs	1
		Young MSM	3
**Regulation**			18
	Endorsement by ministry or NGO^d^		3
	App liability or quality control of medication		1
	Assurance of HIV test kit quality		2
**App developer**			8
	Local versus overseas		4
	Private versus public		2
	University versus company		4

^a^mHealth: mobile health.

^b^N/A: not applicable.

^c^MSM: men who have sex with men.

^d^NGO: nongovernmental organization.

One participant shared the challenges of young individuals (eg, college students) accessing HIV prevention services and indicated the need to tailor the app-based interventions to their specific needs. One potential solution proposed to ensure equitable access in this subgroup was to target college-level LGBTQIA+ (lesbian, gay, bisexual, queer, intersex, asexual, and others) advocacy and support groups to help them access HIV prevention and other health care resources:

They [college students] are often, um, either still under their family’s medical insurance and therefore have difficulty accessing PrEP or have concerns about privacy. So certainly, I think, making the app available to everybody, especially the younger, more vulnerable groups, would be valuable.

As mentioned previously, one of the MSM subpopulations identified by participants as potentially having trouble accessing or using mobile apps for HIV prevention was discreet MSM or those who are not *out* to their respective communities or social circles. It was noted that providing discreet MSM access to such apps could empower them, link them to care, and encourage them to voice their concerns. In addition, 1 participant noted that the app constitutes a way for MSM who use drugs to be potentially linked to HIV care and addiction treatment programs:

But I really hope that those people who are discreet can find an access to start off, you know, like, open up and voice out what is your concern and what is the care you need. That’s why I really hope it [referring to equitable access to MSM subpopulations] can be done.

#### Informed Consent

Several participants indicated the importance of informed consent (mentioned 7 times in total), whereby privacy policies and terms of services must be in place to maximize transparency and clarity between the app developers or owners and the front-end app users (Table 4). More specifically, participants suggested that this feature should be added before users sign up or create an account on the app and include details about the type of data that will be collected and the risks and benefits accompanying the use of the app. Furthermore, participants noted that these documents should ideally cover data collection and storage, whereby users would be able to consent to store their data on the mHealth platform or not:

I think at the beginning of the registration on the app, you must put an option to consent for app use, then it shouldn’t be an issue anymore. So, consent should include gathering data and stuff like that.

#### Regulation

Participants were generally concerned about the regulation and evaluation of the app-based platforms and the services offered via the platform (Table 4). Participants expressed that one of the ways they would use to ascertain the app’s efficacy was to look at the location (ie, overseas or local) of the developers or owners and the type of the institution (ie, private company or academic institution owned) that owned or endorsed the app, with a preference for overseas entities and educational institutions. In addition, participants wanted to ensure that they could trust app developers or the app owner to conduct general quality control and ensure that the services provided through the app were of high quality and from trustworthy sources:

I am more concerned about the medication supply that I receive [through the app]. I would expect that the meds that are being supplied through the apps are legit, through the right source and as well as being approved by the regulators of Malaysia. I expect that this app will review all the medication being supplied.

## Discussion

### Principal Findings and Comparison With Previous Work

mHealth platforms are promising tools that can change the HIV prevention landscape [43-47]. In countries where a hostile sociopolitical environment for MSM prevails, the ensuing stigma and discrimination around homosexuality hinder health care access and have been associated with reduced HIV prevention service uptake among MSM [48,49]. mHealth platforms are well placed to fill this gap, as they provide a web-based platform to deliver HIV services, especially in countries such as Malaysia where MSM are well connected to the internet and use smartphones to seek sexual health information on the web [29]. The findings of this study are critical to assist the development of ethically sound app-based HIV prevention programs, which require the engagement of MSM early on [31].

Among our sample of MSM, concerns about privacy and confidentiality were substantial, bringing forth the issue of third-party data interception, which can occur by accidental disclosure or deliberately through governmental access and subpoena. This finding accentuates previously reported concerns among MSM subgroups regarding the unintentional disclosure of sensitive information to nearby users [31] and further highlights the need for app developers to incorporate app features that address the privacy and confidentiality concerns. Other MSM subgroups have also emphasized their fear of being inadvertently “outed” within their respective communities if they use such platforms in public [50]. Interestingly, a discrepancy between privacy concerns and privacy practices (eg, privacy settings on mobile devices and apps) has also been noted, bringing forth a form of a privacy paradox [51].

In our study, participants stressed on incorporating additional security features in the app (eg, multifactor authentication) and additional features to adjust app visibility, notifications, and avatars to make them more discreet. Similar app features and recommendations were brought up by MSM in previous studies, including clear instructions in the app that prompt users to enable phone privacy features on their mobile device and warnings to view app content in private [52]. More importantly, using encrypted messages, colloquial expressions referring to same-sex behavior, or more neutral content seems to be a recurring suggestion [52,53]. To safeguard user privacy and confidentiality, app developers must communicate with the users about instances in which deidentification of information is not possible and the risk of third-party access to or interception of the data collected via hacking, legal interception, accidental discovery, or telecommunication companies (eg, Google) [33].

Study participants also expressed their concern that access to this platform should be equitable among members of the MSM community, emphasizing the importance of ensuring that specific subgroups, such as hidden MSM and those with low socioeconomic status, have equal access to these services. Similar concerns have been noted in the literature among people living with HIV, whereby ethical considerations such as the accommodation of literacy, infrastructure, and access to technology have been delineated and are viewed as necessary to guarantee equitable access to mHealth services and care [54]. Many established strategies can be implemented to ensure app equity. One potential solution discussed includes the need to market the app to college LGBTQIA+ advocacy groups with greater reach and aptness to share such a platform with vulnerable populations, such as younger MSM, who would be more attentive to privacy risks and, therefore, less able to access such platforms. Adopting a human-centered design approach is also crucial, whereby app developers should increase the participation of participants from underprivileged backgrounds in developing mHealth products to gain insights into their preferences and priorities. In addition to promoting inclusivity by integrating population-specific design or app features, app developers can also promote digital literacy by teaching users how to use mHealth platforms, especially in low-resource or underprivileged subpopulations and settings [55]. Although mHealth technologies are rapidly evolving and despite the impact of sociodemographic inequities on equitable access, there is still a lack of evidence on the equity implications of HIV mHealth platforms. Nevertheless, it is an ethical imperative not to exclude subgroups that lack or have limited access to mHealth, including those who cannot afford to buy a smartphone or have access to the internet and those who might have an impairment that hinders mHealth platform use [33].

A common emerging theme in FGDs was the storage and management of collected data. Participants also referred to entities who will oversee data storage and management, expressing fears regarding data dissemination to third parties (eg, governmental agencies or the public) while maintaining a higher level of trust in general for entities outside of Malaysia as opposed to local entities. MSM’s preferences for eHealth interventions listed in the literature seem to align with these findings, as they expressed fear of disclosure of their HIV status when the intervention originated in organizations or places that they did not know about in the past. In addition, participants would tend to be skeptical about the information disclosed or dismiss messages stemming from such organizations because of the lack of trust [50]. In general, app developers are required to store and transmit the least amount of data possible, which allows the app’s purpose to be fulfilled and to be sensitive to the amount and ways the data are stored on the device itself or transmitted to the clinical team [33]. This ethical consideration has been considered especially sensitive as the data collected are usually detailed and originate from multiple sources that can generate identifiable information if data streams are combined [56]. The unauthorized use of such data can jeopardize user safety and privacy if accessed by third parties [33,56].

Participants emphasized the importance of transparency via privacy policies and terms of service that clearly and explicitly detail which personally identifiable information will be collected, why it is collected, with whom it may be shared, and how users can control their data. It is essential that the participants be given a choice to consent to specific types of data collection and sharing activities [51], which corroborates the concept of “voluntariness” in the context of informed consent, whereby participants should be given a chance to consent to specific data collection streams or app modules but not others [51,56]. However, as there are instances in which opting out of some modules interferes with overall health or risk assessments, the risks and benefits associated with opting in or out of certain app modalities should be thoroughly explained [56]. In addition, using multimodel content, such as audio, video, or infographics, and summarizing key points in laypersons’ terms is encouraged to increase the engagement and comprehension of the users [56-59]. As data protection laws related to mobile apps change over time, these policy documents must be updated, and the users must be informed of such updates accordingly [56,60].

Finally, app-based platforms, particularly when intended to function as aids or alternatives to traditional medical services, carry increased risks to the users. There is a global debate on regulating mHealth apps if they are classified as medical devices. For example, in many high-income countries (eg, the United States and Australia), there are regulatory guidelines that provide oversight to mHealth apps that fulfill the definition of a medical device to ensure safety and effectiveness. However, these do not impede app dissemination, as most mobile apps can still be downloaded by patients via app stores without regulatory filters [33]. Moreover, there are no regulations in many low- and middle-income countries, including Malaysia, that guide app developers on consent, privacy protocols, and ethical practices in delivering health services. Therefore, a dedicated government body must be in place to provide regulatory oversight or “policing” to ensure the safety and effectiveness of such platforms. In addition, mHealth app developers must maintain transparency regarding the scientific evidence (or lack of evidence) underpinning the app’s effectiveness.

### Limitations and Strengths

Our study has some limitations. First, our sample of MSM excludes those who are not active on GSN apps, do not have access to the internet, and do not read or understand English or Bahasa Malaysia. Furthermore, although theoretical saturation was reached after the third session, it is likely that additional ethical perceptions that were not brought up during the FGDs exist, which could be identified, for example, by recruiting participants in ways that differ from ours (ie, GSN app advertisements and direct profile inquiries). Second, most participants in our sample were of Chinese ethnicity, had tested for HIV, and were aware of PrEP, which limits the generalizability of our findings to the broader MSM group in Malaysia. Enrolling predominantly Malay participants or MSM who were not linked to HIV prevention services could yield different perspectives on ethical issues. Nevertheless, this study is among the first to examine the ethical issues surrounding the use of smartphone apps for HIV prevention among MSM in Malaysia, which is the first step toward understanding and engaging this specific subgroup. In addition, the web-based modality of the FGDs constituted a safe, confidential, and easily accessible platform for participants, thereby creating a safe environment and fostering a higher level of truthfulness among participants.

### Conclusions

In countries such as Malaysia, where homosexuality is illegal, stigma and discrimination around HIV make traditional venue-based HIV prevention services less suitable for vulnerable populations, such as MSM. The findings from this study indicate the potential implications of ethical concerns and associated risks related to using app-based HIV prevention programs in low- and middle-income countries such as Malaysia. Such platforms can be kept safe by integrating security features (eg, multifactor authentication and discreet features to adjust app or notification visibility); storing and transmitting the least amount of data possible; ensuring transparency via clear privacy policies and terms of service; giving participants a choice to consent to specific types of data being collected; and, in the case of medical devices, ensuring adequate regulation by dedicated government bodies. Our findings indicate the need for the systematic engagement of all relevant stakeholders (eg, MSM, community members, and health care providers) and the adoption of an ethical framework in the design and development phases to ensure that the platform is ethical, safe, secure, equitable, and sustainable.

## Abbreviations

FGD: focus group discussion
GSN: geosocial networking
LGBTQIA+: lesbian, gay, bisexual, queer, intersex, asexual, and others
mHealth: mobile health
MSM: men who have sex with men
PrEP: pre-exposure prophylaxis

## Data Availability

The data sets generated or analyzed during this study are available from the corresponding author on reasonable request.
